# Effects of pretreatment on morphology, chemical composition and enzymatic digestibility of eucalyptus bark: a potentially valuable source of fermentable sugars for biofuel production – part 1

**DOI:** 10.1186/1754-6834-6-75

**Published:** 2013-05-09

**Authors:** Marisa A Lima, Gabriela B Lavorente, Hana KP da Silva, Juliano Bragatto, Camila A Rezende, Oigres D Bernardinelli, Eduardo R deAzevedo, Leonardo D Gomez, Simon J McQueen-Mason, Carlos A Labate, Igor Polikarpov

**Affiliations:** 1 Instituto de Física de São Carlos, Universidade de São Paulo, Caixa Postal 369, 13560-970, São Carlos, SP, Brazil; 2 Laboratório Max Feffer de Genética de Plantas, Departamento de Genética - ESALQ, Universidade de São Paulo, Caixa Postal 83, 13418-900, Piracicaba, SP, Brazil; 3 CNAP, Department of Biology, University of York, Heslington, York YO10 5YW, UK

**Keywords:** *Eucalyptus grandis*, *Eucalyptus grandis* x *urophylla*, Bark, Bioethanol, Acid pretreatment, Alkali pretreatment, Chemical composition, Solid-state NMR, FTIR, Scanning electron microscopy, Enzymatic hydrolysis

## Abstract

**Background:**

In recent years, the growing demand for biofuels has encouraged the search for different sources of underutilized lignocellulosic feedstocks that are available in sufficient abundance to be used for sustainable biofuel production. Much attention has been focused on biomass from grass. However, large amounts of timber residues such as eucalyptus bark are available and represent a potential source for conversion to bioethanol. In the present paper, we investigate the effects of a delignification process with increasing sodium hydroxide concentrations, preceded or not by diluted acid, on the bark of two eucalyptus clones: *Eucalyptus grandis* (EG) and the hybrid, *E. grandis* x *urophylla* (HGU). The enzymatic digestibility and total cellulose conversion were measured, along with the effect on the composition of the solid and the liquor fractions. Barks were also assessed using Fourier-transform infrared spectroscopy (FTIR), solid-state nuclear magnetic resonance (NMR), X-Ray diffraction, and scanning electron microscopy (SEM).

**Results:**

Compositional analysis revealed an increase in the cellulose content, reaching around 81% and 76% of glucose for HGU and EG, respectively, using a two-step treatment with HCl 1%, followed by 4% NaOH. Lignin removal was 84% (HGU) and 79% (EG), while the hemicellulose removal was 95% and 97% for HGU and EG, respectively. However, when we applied a one-step treatment, with 4% NaOH, higher hydrolysis efficiencies were found after 48 h for both clones, reaching almost 100% for HGU and 80% for EG, in spite of the lower lignin and hemicellulose removal. Total cellulose conversion increased from 5% and 7% to around 65% for HGU and 59% for EG. NMR and FTIR provided important insight into the lignin and hemicellulose removal and SEM studies shed light on the cell-wall unstructuring after pretreatment and lignin migration and precipitation on the fibers surface, which explain the different hydrolysis rates found for the clones.

**Conclusion:**

Our results show that the single step alkaline pretreatment improves the enzymatic digestibility of Eucalyptus bark. Furthermore, the chemical and physical methods combined in this study provide a better comprehension of the pretreatment effects on cell-wall and the factors that influence enzymatic digestibility of this forest residue.

## Background

The search for new technologies aimed at the production of renewable biofuels has intensified in recent years. There is an increasing world-wide interest in the limitation of environmental impacts and climate changes by replacing petrochemical products with environment-friendly analogues in order to move towards a sustainable economy [1,2]. Fossil fuels are being replaced by alternative fuels from renewable sources all over the world [3]. Since the 1970s, Brazil started a program to replace gasoline by ethanol produced from sugarcane juice that has led to 90% of vehicles within the country being fuelled in this way. More recently, the European Union has adopted a mandate compelling each member country to substitute 10% of all transport fuels for biofuels by 2020 [2,4]. At present, bioethanol is mainly produced from food sources, such as seeds, grains, or sugarcane juice leading to criticisms regarding competition between food and fuels for agricultural resources. Given this scenario, bio-refining biomass to produce several products such as fuels and other biomaterials from the same feedstock has become a vibrant research area.

Eucalyptus, poplar and pine are commercially important fast-growing trees that are widely used in the construction sector as well as the pulp and paper industries [5]. Eucalyptus plantations can be found in more than 90 countries on five continents and is by far the fastest-growing hardwood forestry industry in the world, with a total plantation area estimated at between 16 and 19 million hectares (40–47 million acres) [6]. Most of the current Eucalyptus production is in South America (over 55% of the worldâ€™s Eucalyptus roundwood). In 2010, Brazil had around 4.8 million hectares, almost a third of the global Eucalyptus plantation area [6,7]. Annual Brazilian standing timber productivity in short-rotation Eucalyptus plantations can reach 40–80 m^3^/ha/year (over bark), whereas in other regions of the world it is around 25 m^3^/ha/year [8]. Eucalyptus production and processing generates a large amount of wood residues, such as bark and branches, which are currently left in the field to enrich the soil. The proportion of residues can reach 30% of the total biomass harvested (15–25 ton/ha/year), whereas 10-12% of all this volume is bark [9-12]. The high amount of bark available and the fast-growth of eucalyptus trees make this agricultural residue a promising feedstock for bioethanol production.

The main differences between eucalyptus residues and other agricultural residues are their physical properties and chemical composition. In general, hardwood biomass, such as eucalyptus, has a considerably lower content of pentose sugars compared to cereal straws or biomass grasses. This is an advantage for bioethanol production, since pentose fermentation to ethanol is unfavorable when using yeast. However, eucalyptus wood and barks are harder and denser than grass or cereal biomass, and, as a result of its higher lignin content, it is more recalcitrant to microbial and enzymatic action [11,13]. Because of this, different pretreatment and saccharification conditions are likely to be needed, compared to those used with other feedstock.

Only a few studies about the acid and enzymatic hydrolysis of Eucalyptus biomass to biofuel production have been published [5,14-20]. However, most of them have focused on wood or mixed harvesting residues (branches, leaves and barks) conversion using diluted acid, organosolv and hydrothermal pretreatment. Canettieri and co-workers [20] used diluted sulfuric acid as a catalyst for hydrolysis of *Eucalyptus grandis* residues, mainly focused on hemicellulose removal and consequently on the production of fermentable sugars (xylose, glucose and arabionose) as well as on the by-products formation (furfural, 5-hydroxymethylfurfural and acetic acid). Pretreatment and enzymatic digestibility of Eucalyptus barks were described by Matsushita et. al. (2010) [21], who evaluated the hydrothermal pretreatment with carbon dioxide for enhancing barks saccharification.

In this paper, we describe for the first time the potential of the bark from two commercial eucalyptus clones widely cultivated in Brazil, *E. grandis* (EG) and a hybrid between *E. grandis* x *E. urophylla* (HGU) for biofuel production, using a one or two-step pretreatment method with increasing NaOH concentrations, preceded (or not) by a dilute acid treatment. We also investigated the changes in the morphology and crystallinity of the eucalyptus barks and their relation to chemical composition and enzymatic hydrolysis efficiency.

## Results and discussion

### Determination of eucalyptus bark composition

Chemical composition was determined for the raw bark of *E. grandis* and *E. grandis x urophylla*, and is presented in Table 1. The main monosaccharides found in *Eucalyptus* bark were glucose (approximately 39% and 40% in HGU and EG, respectively), followed by xylose (approximately 10% in HGU and 9% in EG). This is consistent with the previously published results. For example, Yu et al. (2010) found 44.9% and 11.4% of glucan and xylan, respectively, in *E. grandis* residues (branches, leaves and barks) [5]. Low amounts of fucose, *rhamnose*, arabinose and galactose were also detected. In addition, EG and HGU barks have around 5.2% and 9.2% of soluble sugars respectively. This is associated with the physiological role of bark as the site of phloem transport and it also forms part of the cambial tissues, which have high sugar requirements. The tree barks consist mainly of three types of cells—phloem fibers, sieve cells, and phloem parenchyma cells, which are responsible for conducting the nutrients along the plant [21]. Thus, the eucalyptus barks, HGU and EG, could represent an important source of soluble sugars in addition to cellulosic sugars for bioethanol production.

**Table 1:** **Chemical composition of bark from two commercial clones, *Eucalyptus grandis *(EG) and *Eucalyptus grandis *x*urophylla *(HGU)**

**Cell-wall monosaccharides (%)**	**HGU**	**EG**
**Fucose**	0.10 ± 0.01	0.12 ± 0.01
**Rhamnose**	0.32 ± 0.04	0.34 ± 0.04
**Arabinose**	1.03 ± 0.04	1.14 ± 0.04
**Galactose**	0.91 ± 0.03	1.19 ± 0.03
**Glucose**	38.85 ± 0.90	39.55 ± 0.66
**Xylose**	9.62 ± 0.10	8.64 ± 0.15
** Total solubles (%)**	25.77 ± 0.82	26.64 ± 0.73
*** Soluble Sugars (%)***		
***Glucose***	*1.71 ± 0.17*	*0.76 ± 0.04*
***Fructose***	*4.54 ± 0.58*	*2.60 ± 0.15*
***Sucrose***	*2.90 ± 0.28*	*1.81 ± 0.07*
** Total lignin (%)**	19.68 ± 0.33	14.71 ± 0.34
***Klason Lignin***	*16.86 ± 0.41*	*11.41 ± 0.45*
***Soluble lignin***	*2.82 ± 0.36*	*3.30 ± 0.20*
** Ashes**	4.06 ± 0.04	7.14 ± 0.25
** Total (%)**	**100.34 ± 4.16**	**99.47 ± 3.78**

Amounts are expressed in percentage of a dry matter.

The total soluble fraction obtained by extraction under conditions of increasing polarity was 25.8% for HGU and 26.7% for EG bark. According to previous published data, the soluble amount on the inner bark of *E. globulus* is around 20.6%, using a sequential extraction with ethanol:benzene (1:2 v/v) and 70% aqueous acetone [21]. The major soluble extractives on the eucalyptus bark are mainly composed of tannins, polyphenolic compounds, fatty acids and flavonoids, and the amount of which have a significant influence on bioethanol yield since some may act as inhibitors during fermentation [22].

The total lignin content of raw bark was determined by measuring the Klason and the soluble lignin, as shown in Table 1. The total lignin found for HGU was 19.7%, while for EG bark this value was 14.7%. Matsushita and co-workers have found around 12% of Klason lignin on the inner bark of *E. globulus*[21], similar to the value encountered for EG bark (11.4%), while HGU had around 16.8% of Klason lignin (Table 1). Ash content was 4.1% for HGU and 7.1% for EG. The inorganic fraction of eucalyptus barks is manly composed of calcium crystals in the form of calcium oxalate or carbonate, whereas lignocellulosic materials from grasses contain mainly silica [12,23]. X-ray fluorescence showed that the bark from both eucalyptus clones contain a silicon level around 0.03% ± 0.01, while sugarcane bagasse presents about 0.45% ± 0.03 (data not shown).

**Figure 1:**
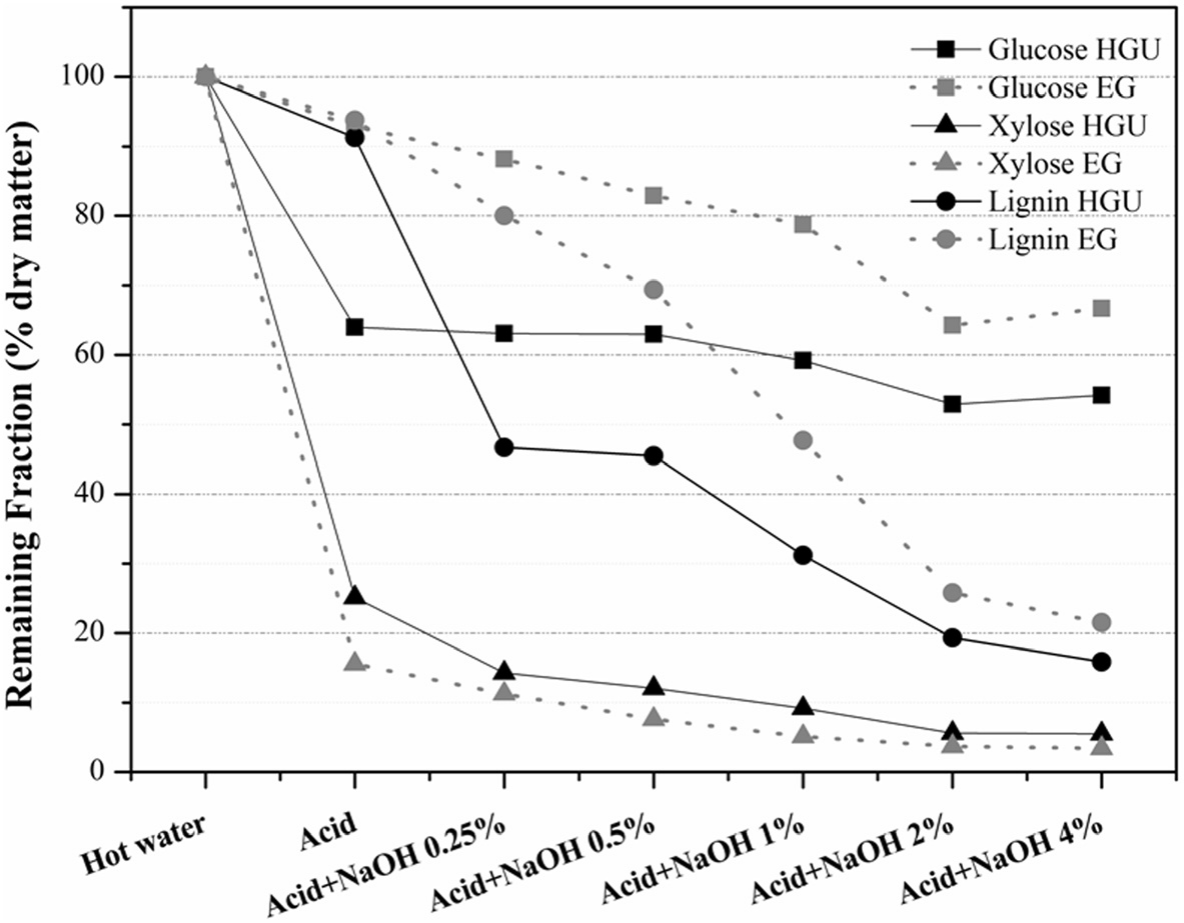
Remaining glucose, xylose and total lignin content in the solid fraction from HGU and EG barks after each pretreatment step.

Prior to the pretreatment steps, the bark underwent extraction with hot water (80°C, for 1 h) to remove any extractives and soluble sugars. Previous reports showed 60% of extractives are removed from eucalyptus bark using only hot water [22]. The main monosaccharides (glucose and xylose) and total lignin content for water extracted bark as well as after different pretreatment conditions are shown in Table 2. Lignin, xylose and glucose percentages increase after pretreatment due to the removal of soluble sugars and extractives. Table 2 and Figure 1 both show that acid treatment affects mainly the hemicellulose fraction, removing around 75% in HGU and 85% in EG. Part of the lignin fraction was also removed by the acid treatment (8.7% for HGU and 6.2% for EG), although hemicellulose is the main biomass component removed. Thus, the total lignin content increased to *ca.* 32% for both clones after acid pretreatment. Table 3 shows that around 65% and 59% of soluble lignin was removed for HGU and EG, respectively, in contrast with the small effect of acid pretreatment on Klason lignin.

**Table 2:** Quantification of the main components (total lignin, xylose and glucose) of eucalyptus bark (HGU an EG clones) after hot water extraction and each pretreatment step

**Treatment**			**Cell-wall components (%)**							
	**Liquor fraction**				**Monosaccharides**					
	**5-HMF (mg/g bark)**		**Total lignin**		**Xylose**		**Glucose**		Treatment yield*	
	**EG**	**HGU**	**EG**	**HGU**	**EG**	**HGU**	**EG**	**HGU**	**EG**	**HGU**
**Hot Water**	-	-	25.1 ± 0.3	27.1 ± 0.6	15.0 ± 0.8	12.0 ± 0.2	41.5 ± 10.1	54.5 ± 11.7	100,0	100,0
**Acid 1%**	0.20 ± 0.05	0.23 ± 0.03	32.0 ± 0.3	32.0 ± 0.4	3.3 ± 0.5	4.2 ± 1.0	54.8 ± 14.0	47.2 ± 5.4	92,9	64,0
**Acid 1% + NaOH 0.25%**	0.03 ± 0.01	0.03 ± 0.01	30.7 ± 2.2	22.3 ± 0.6	2.4 ± 0.3	3.1 ± 0.4	58.6 ± 14.3	63.2 ± 1.6	88,2	63,1
**Acid 1% + NaOH 0.5%**	-	-	28.1 ± 0.3	22.6 ± 1.4	1.9 ± 0.2	2.7 ± 0.5	64.9 ± 6.1	66.5 ± 10.6	82,9	63,0
**Acid 1% + NaOH 1%**	-	-	23.9 ± 0.2	18.3 ± 0.3	1.6 ± 0.3	2.5 ± 0.3	66.5 ± 10.5	72.2 ± 10.7	78,7	59,2
**Acid 1% + NaOH 2%**	-	-	17.0 ± 0.2	13.0 ± 0.2	1.5 ± 0.5	1.7 ± 0.4	68.9 ± 3.3	73.4 ± 3.0	64,3	52,9
**Acid 1% + NaOH 4%**	-	-	14.7 ± 0.2	11.6 ± 0.1	1.8 ± 0.2	1.4 ± 0.2	75.8 ± 6.7	81.15 ± 1.5	66,7	54,2
**NaOH 4%**	-	-	19.3 ± 0.3	18.4 ± 0.6	12.0 ± 3.3	13.3 ± 3.8	56.1 ± 17.0	61.8 ± 12.3	73,1	63,4

Amounts are expressed in percentage of dry matter.

*Treatment yield (%) is calculated based on glucose losses (as a percentage of dry mass) during each pretreatment step, considering the initial amount in a hot water extracted sample. For samples submitted to a double-step pretreatment we considered the losses from acid plus alkaline steps.

**Table 3:** Quantification of soluble and Klason lignin of eucalyptus barks (HGU an EG clones) after hot water extraction and each pretreatment step

**Treatment**	**Lignin content**					
	**EG**		**HGU**		**Total lignin (%)**	
	**Soluble (%)**	**Klason (%)**	**Soluble (%)**	**Klason (%)**	**EG**	**HGU**
**Hot Water**	1.7 ± 0.1	23.4 ± 0.6	2.9 ± 0.9	24.2 ± 0.5	25.1 ± 0.3	27.1 ± 0.6
**Acid 1%**	0.7 ± 0.1	31.3 ± 0.5	1.0 ± 0.3	31.0 ± 0.7	32.0 ± 0.3	32.0 ± 0.4
**Acid 1% + NaOH 0.25%**	0.5 ± 0.2	30.2 ± 1.5	0.6 ± 0.2	21.7 ± 0.8	30.7 ± 2.2	22.3 ± 0.6
**Acid 1% + NaOH 0.5%**	0.6 ± 0.2	27.5 ± 0.6	0.5 ± 0.2	22.1 ± 1.3	28.1 ± 0.3	22.6 ± 1.4
**Acid 1% + NaOH 1%**	0.6 ± 0.3	23.3 ± 0.5	0.7 ± 0.3	17.6 ± 0.6	23.9 ± 0.2	18.3 ± 0.3
**Acid 1% + NaOH 2%**	0.7 ± 0.1	16.3 ± 0.5	0.5 ±0.1	12.5 ± 0.5	17.0 ± 0.2	13.0 ± 0.2
**Acid 1% + NaOH 4%**	0.4 ± 0.2	14.3 ± 0.5	0.5 ± 0.2	11.1 ± 0.3	14.7 ± 0.2	11.6 ± 0.1
**NaOH 4%**	1.7 ± 0.5	17.6 ± 0.7	2.2 ± 0.6	16.2 ± 0.9	19.3 ± 0.3	18.4 ± 0.6

Amounts expressed as percentage of dry matter.

The yields after treatment based on glucose losses (in percentages of dry matter) are indicated in Table 2. The cellulose fraction in HGU bark presents high susceptibility to acid pretreatment, since approximately 36% of the total glucose was removed during this step. This could be a consequence of the higher content of amorphous cellulose in HGU compared to EG bark, where only 7% of glucose is removed (Table 2 and Figure 1). The analysis of the pretreatment liquor from the acid pretreatment reinforces the differential degradation of the cellulosic fraction. High levels of 5-HMF were detected in the acid liquor from both barks (0.20 ± 0.05 and 0.23 ± 0.03 mg/g bark for HGU and EG, respectively; Table 2). Considering the alkaline liquor fraction, low levels of 5-HMF (0.03 ± 0.01 mg/g for both clones) were detected only at 0.25% NaOH concentration. Since 5-HMF was not detected in the liquor fraction obtained from the treatment with 4% NaOH only, it suggests that 5-HMF comes from the acid pretreatment step. Furthermore, it is conceivable that the alkaline step, at concentrations higher than 0.25% of NaOH, has worked as an efficient detoxification step. Indeed, the removal of inhibitors, such as furfural and 5-HMF, by neutralization using NaOH or Ca(OH)2 has been already described [24,25]. Nilvebrant and collaborators reached a 20% reduction in concentration of furfural and hydroxymethylfurfural in diluted-acid hydrolizate of spruce, when pH was adjusted to 10 using NaOH and Ca(OH)2 [26]. Since the temperature used in this work was only 120°C, a small amount of furfural (35 μg/g bark) was found only in the EG liquor fraction from acid pretreatment. Previous studies have shown low or no formation of hemicellulose degrading products, such as furfural, at temperatures below 160°C for acid pretreatment [27,28]. The effects of alkaline pretreatment that was implemented after the acid step are presented in Table 2. Pretreatment with 4% NaOH mainly affected the lignin fraction, removing 84.1% and 78.5% of total lignin for HGU and EG barks, respectively (Figure 1). The lignin fraction was gradually removed with increasing NaOH concentrations, reaching 11.1% for HGU and 14.3% for EG, under the harshest pretreatment conditions. The residual hemicellulose fraction was also removed to levels lower than 2% of total dry matter. The glucose content was significantly increased after treatment with acid and 4%NaOH, attaining around 81% of dry weight matter for HGU and 78% for EG. However, the glucose losses were 45.8% and 33.3% for HGU and EG, respectively, after acid plus 4% NaOH treatment (Table 2 and Figure 1). These results are similar to those previously published by our research group on sugarcane bagasse using the same pretreatment conditions [29].

Eucalyptus barks were treated with 4% NaOH, without the initial acid treatment in order to evaluate the need for the first acid step to enhance the effects of pretreatment and its impacts on the enzymatic hydrolysis yields. The glucose fraction reached 62% in HGU and 56% in EG barks, followed by low glucoses losses (36.6% and 26.9% for HGU and EG, respectively) with no 5-HMF generation in the liquor fraction (Table 2). The total hemicellulose removal was around 40% and 56%, while the total lignin fraction decreased 63.7% and 59%for HGU and EG, respectively in dry bark.

#### FTIR-PCA

FTIR spectral data is widely used for chemical analysis of pulp and wood [30,31]. We applied this technique to HGU and EG bark after each pretreatment condition and differences between the hot water extracted and pretreated samples were analyzed in the spectral region between 850–1850 cm^-1^. Characteristic assignment of hemicellulose at 1738/1734 cm^-1^ (C = O conjugates in xylans) was only observed in hot water treated bark for both HGU and EG [32]. In all the pretreatment conditions, this peak was absent, confirming the efficiency in removing hemicellulose (data not shown). A gradual decrease in intensities in the regions comprising the aromatic ring vibration and the C = O stretch around 1600 cm^-1^ as well as the aromatic skeletal vibration in lignin at 1505/1511 cm^-1^ were also evident for both bark types [33,34]. Small differences in the intensity of the peak at 1375 cm^-1^ related to the C-H deformation in cellulose and hemicellulose were observed, while a significant decrease of the peak at 1325 cm^-1^ was detected (C-H vibration in cellulose and C_1_-O vibration in syringyl derivatives) as a function of increasing NaOH concentration (data not shown) [32]. The heteropolymeric lignin macromolecule is derived mainly from the polymerization of three types of hydroxycinnamic alcohol monomers: guaiacyl (coniferyl alcohol), syringyl (sinapyl alcohol), and *p-*hydroxyphenyl (hydroxycinnamyl alcohol), and their proportions can be vary for different groups of plants [35]. As an angiosperm, the lignin fraction in the eucalyptus is composed by guaiacyl-syringyl monomers, while in gramineae such as sugarcane bagasse, a mixture of all three monomers can be found [36]. Low intensities were detected at around 1268 and 1230 cm^-1^, which can be assigned to guaiacyl derivatives (C-O stretch in lignin and C-O linkage in guaiacyl aromatic methoxyl groups) [32]. However, a well-defined peak around 1030 cm-1 with a high intensity at around 1030 cm-1, which is also attributed to guaiacyl moieties, was detected and gradually removed with increasing NaOH concentrations [32]. A similar gradual decrease in intensity was also observed for the peak at 1122 cm-1, which is assigned to aromatic skeletal and C-O stretch [37]. By increasing the NaOH concentration, a higher definition of cellulose peaks (1170 cm-1 and 898 cm-1) became evident, which is a result of the removal of the hemicellulose and lignin groups [37].

**Figure 2:**
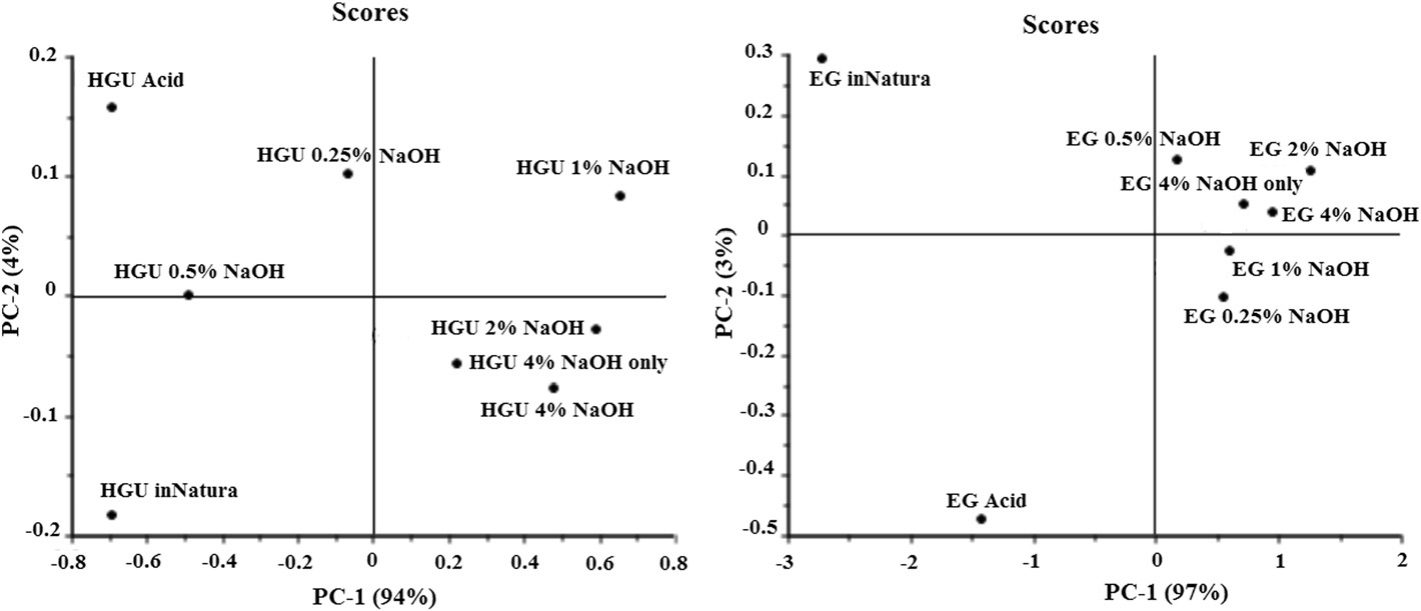
FTIR-PCA scores plot of PC-1 and PC-2 obtained for HGU and EG eucalyptus clones bark undergoing different pretreatment conditions.

**Figure 3:**
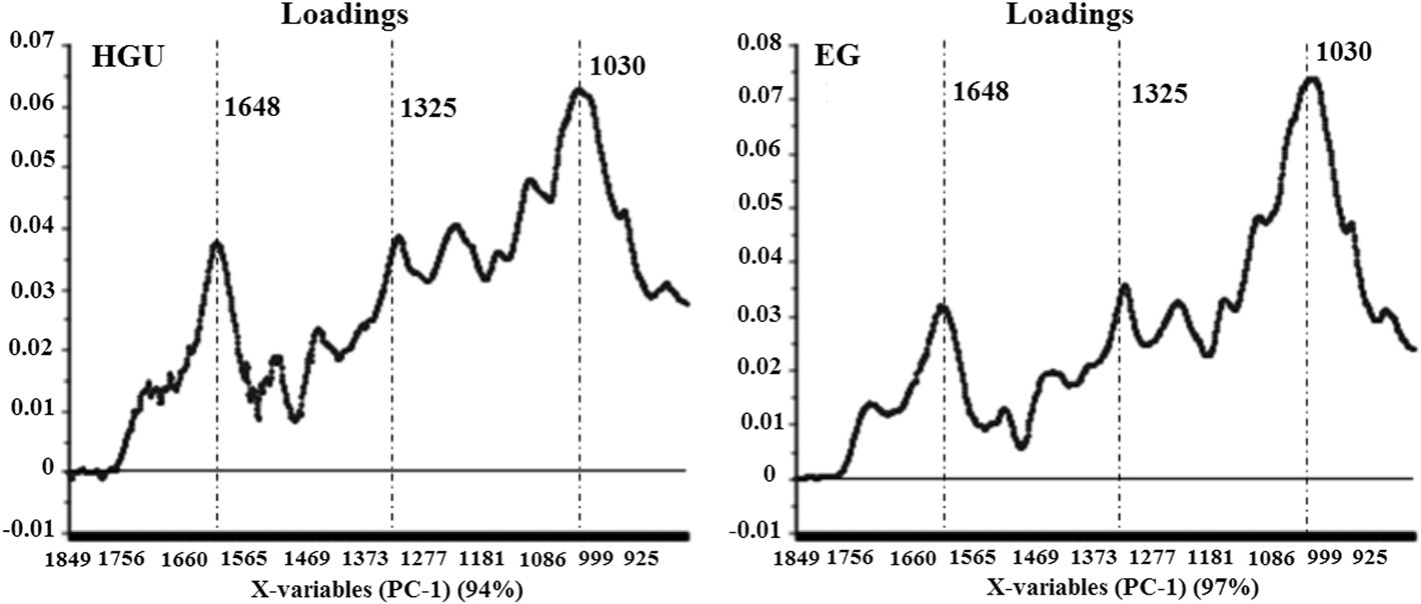
Loadings of PC-1 of FTIR of HGU and EG eucalyptus clones bark.

Despite the differences reported above, the univariate analysis of FTIR spectra for biomass is insufficient to obtain accurate information to evaluate complex changes [33]. Therefore, the effects of pretreatment were investigated using principal component analysis (PCA; Figures 2 and 3) of the total spectrum. The results show that the first two principal components (PC) explained 98% and 100% of the variance between the samples for HGU and EG, respectively (Figure 2). According to the score plots, the hot water and the acid treated samples are significantly different from each other in both eucalyptus barks, presenting an opposite arrangement along PC-2. The main chemical difference between these samples is the hemicellulose content (Table 2 and Figure 1), suggesting that PC-2 is associated to the hemicellulose signals (which is responsible for 4% and 3% of the samples variance in HGU and EG, respectively). PC-1 on the other hand can be mainly related to lignin changes, explaining above 90% of the variance between the samples (94% for HGU and 97% for EG). It is also clear in the score plots that HGU samples are significantly different up to a concentration of 1% NaOH. Samples treated with 2% NaOH or higher (with or without a previous acid step) do not separate in the PCA analysis. On the other hand, EG samples treated with any concentration of NaOH can be grouped together along the positive side of PC-1. This arrangement of the NaOH pretreated samples of EG can be associated to a more gradual removal of lignin and glucose as showed in Figure 1.This is the opposite to what is observed for HGU, which presented a sharp lignin removal between acid and acid plus 0.25% NaOH, as well as between 0.5% and 2% NaOH. HGU also had an almost constant glucose content after acid treatment (Figure 1).

Figure 3 shows the loadings responsible for the scores along PC-1 for EG and HGU. The PC-1 loading plot is complex, suggesting that the scores result from the changes concerning different groups of bonds. It confirms, however, that the main loadings observed for PC-1 in both clones are located at 1648, 1325 and 1030 cm^-1^, which are related to lignin signals (O-H and conjugated C-O; C-H vibration in cellulose and C-O vibration in syringyl derivatives; and guaiacyl moieties, respectively).

#### Solid-state NMR analysis

**Figure 4:**
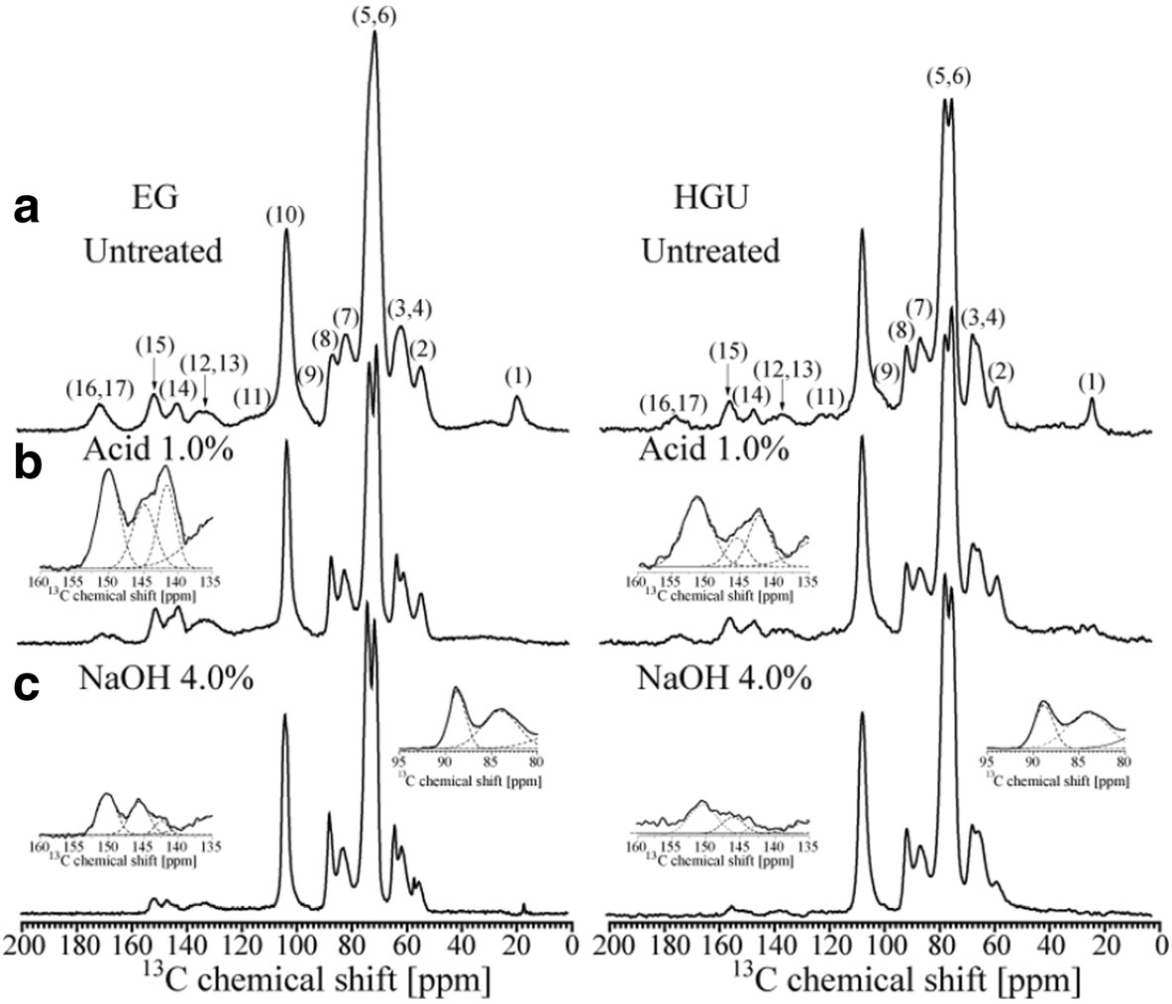
CPMASTOSS NMR spectra of EG and HGU bark samples without pretreatment (a), after acid (b) and alkaline (NaOH 4%) (c) pretreatments.

Figure 4 shows CPMASTOSS spectra of the solid fractions of hot water extracted eucalyptus barks and pretreated samples. The chemical shift assignments are based on the comparison of the ^13^C NMR spectra from the bark samples and sugarcane bagasse using the same pretreatment [29,38,39].

In Figure 4, the peaks in the 50 to 120 ppm region are assigned to cellulose carbons with contributions also from hemicellulose and lignin signals for both untreated EG and HGU samples. Peak 3 at 62.6 ppm and peak 7 at 84.0 ppm are assigned, respectively, to C6 and C4 carbon from amorphous cellulose, while peaks 4 at 65.0 ppm and 8 at 88.9 ppm are assigned to C6 and C4 carbon in crystalline cellulose [40-44]. Lignin signals are spread throughout the spectral region, but the peaks labeled as 2, 11,12,13,14 and 15 are specific to lignin (Table 4) [38,39]. Hemicellulose carbons contribute to peaks 1, 3, 6, 7, 9 and 17 [29,38,39].

**Table 4:** Assignments of NMR lines 1 to 17

**Line number**	**Chemical group**	^**13**^**C chemical shift [ppm]**
1	CH_3_ in acetyl groups of hemicelluloses	22
2	Aryl methoxyl carbons of lignin	56
3	C6 carbon of non-crystalline cellulose	63
4	C6 carbon of crystalline cellulose	65
5	C2,3,5 of cellulose, OC_α_H_2_ carbons lignin	73
6	C2,3,5 of cellulose and hemicelluloses	75
7	C4 carbon of non-crystalline cellulose	84
8	C4 carbon of crystalline cellulose	88
9	Shoulder of C1 carbon of hemicelluloses	102
10	C1 carbon of cellulose	105
11	C2 and C6 aromatic carbons of syringyl and C5 and C6 aromatic carbons of guaiacyl in lignin	110-115
12	C2 of aromatic carbons guaiacyl in lignin	127
13	C1 and C4 aromatic carbons of syringyl (e) and (ne)	136
14	C3 and C5 aromatic carbons of syringyl (ne) and C1 and C4 aromatic carbons of guaiacyl in lignin	148
15	C3 and C5 aromatic carbons of syringyl (e) in lignin	154
16	Carboxyl groups of lignin	163-180
17	Carboxyl groups of hemicelluloses	174

In the spectra of acid treated EG and HGU solid fractions (Figure 4b) a visible enhancement of the spectral resolution in the 50 to 120 ppm region is observed together with a decrease in intensity of peaks 1 and 17, which are attributed to the removal of hemicellulose. A better spectral resolution is observed for the untreated HGU sample when compared to EG. This suggests that the initial amount of hemicellulose is greater in the later. This is also reinforced by the split of a single peak into two peaks, 5 and 6, in untreated HGU as well as the higher intensity (integral) of peak 1 for the untreated EG (Figure 4a).

**Figure 5:**
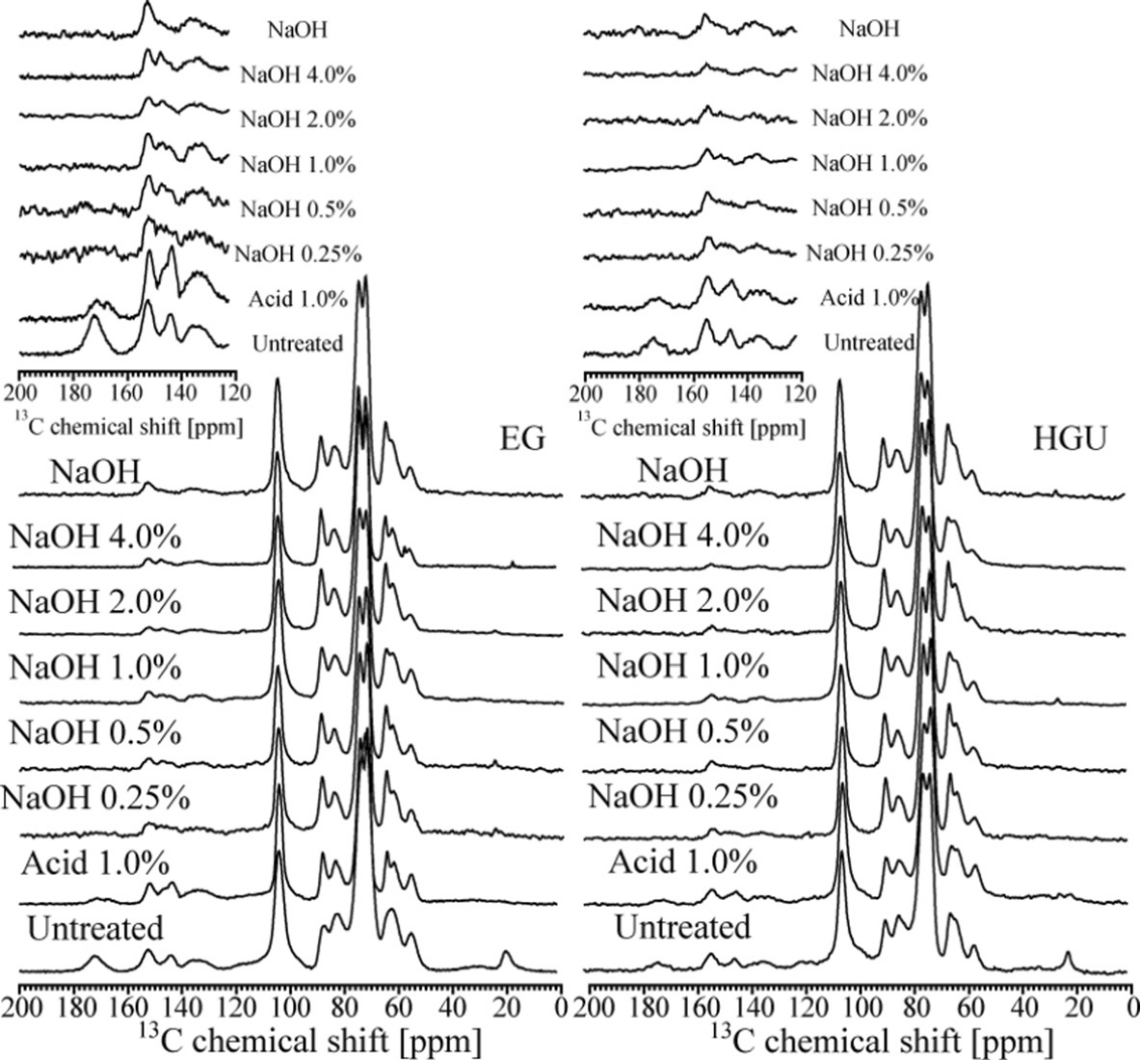
CPMASTOSS NMR spectra of eucalyptus bark samples after and before pretreatment.

The spectra of EG and HGU solid fractions treated with 4% NaOH after acid treatment are shown in Figure 4c. As mentioned previously, the peaks at 84.0 and 88.9 ppm are due to the amorphous and crystalline cellulose. Thus, comparing the spectra between Figure 4b and 4c, there is an increase in crystalline compared to amorphous cellulose, as reported previously for the pretreatment of other hard and soft woods [29,45]. The percentage of crystalline and amorphous cellulose can be estimated from the ratio between each peak intensity (area under the crystalline or amorphous signal) and the full intensity (crystalline + amorphous). This method generally gives a lower crystallinity index than the XRD methods, since NMR takes into account the cellulose chains present on the surface of cellulose crystals [46]. Eucalyptus bark samples produce signal overlaps between lignin, hemicellulose and amorphous cellulose, thereby resulting in lower estimates of the percentage of crystalline cellulose present. To obtain a better estimation of the peak intensities this spectral region was fitted with two Gaussian peaks and the result is shown as an inset in Figure 4c. The crystalline cellulose was found to be 41% and 36% for EG and HGU samples, respectively, when taking the two Gaussian areas into account. Since all spectra were normalized with respect to peak 10 (C1 carbon of cellulose), the decrease in the intensities of peaks 2 and 11–15 (Figure 4c) indicates a significant reduction of the lignin to cellulose fraction upon the treatment with NaOH. This is in line with the results obtained from the chemical composition analysis and FTIR. The CPMASTOSS spectra of the NaOH treated samples show a progressive decrease of lignin signals upon increasing NaOH concentration. Relative intensities of the lignin signals in the samples treated with 4% NaOH are lower in HGU than in EG, thus revealing that the alkaline treatment was more efficient for HGU (refer to the insert of the amplified region from 120 to 200 ppm in Figure 5). This is in line with the changes observed in the chemical composition reported in Table 2.

Interestingly, the CPMASTOSS spectra of EG and HGU samples treated only with 4% NaOH are quite similar to that of the samples treated with 1% HCl plus 4% NaOH treatment. This confirms that the NaOH treatment at higher concentration results in the removal of both hemicellulose and lignin, which is in agreement with our chemical analysis. Other interesting effects shown in Figure 5 are the different decreasing rates of the signals in the 140 to 160 ppm region. This spectral region can be fitted by three Gaussian lines with a chemical shift of 153.0, 148.0 and 144.6 ppm. According to Martinez and co-workers the signal at 153.0 ppm arises from the C3 and C5 aromatic carbons of syringyl (etherified) units in lignin, the signal at 148.0 ppm is due to C3 and C4 aromatic carbons of syringyl (phenolic) and to C3 and C5 aromatic carbons of guaiacyl (phenolic) units in lignin [47]. There is also a strong signal at 144.6 ppm that has been previously assigned to polyphenolic compounds condensed within lignin, which are responsible by the reddish color of some woods, such as *E. cordifolia*[47-50]. The intensity of the 144.6 ppm signal progressively decreased and the color of the samples changed from dark red to dark yellow (data not shown) as the NaOH concentration was increased. The higher intensity ratio between the signals at 153.0 and 148.0 ppm in the HCl treated EG sample is indicative that this sample has a higher amount of syringyl units in lignin compared to HGU [47].

**Figure 6:**
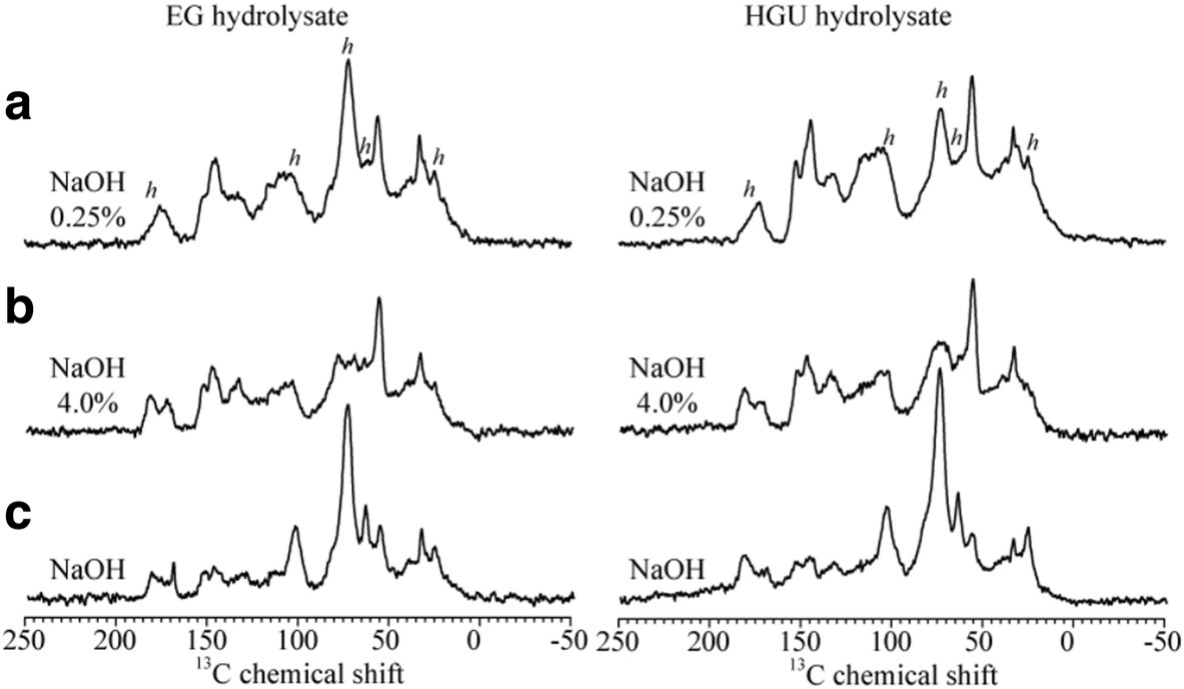
CPMASTOSS NMR spectra of lyophilized hydrolysates (liquor fraction) from the EG and HGU treated with NaOH concentrations of 0.25% (a) and 4.0% (b) after acid treatment, and only 4% NaOH (c).

Figure 6 shows the CPMASTOSS spectra of lyophilized hydrolysates (liquor fraction) from EG and HGU treated with NaOH concentrations of 0.25% or 4.0% after acid treatment, as well as with 4% NaOH. The presence of the hemicellulose signals after NaOH treatment (indicated by an *h* in Figure 6a) is due to the removal of the hemicellulose that remain after the acid treatment. In Figure 6a, the HGU hydrolysate lignin signals are higher than those of hemicellulose, while in the EG hydrolysate the opposite is observed. This is in agreement with the higher amount of hemicellulose found in the EG sample. The lignin signals are predominant in the hydrolysate resulting from the 4% NaOH treatment, which suggests that the hemicellulose can be removed at lower NaOH concentrations, but the removal of the lignin requires higher concentrations of NaOH. The results in Figure 6c also confirm that the NaOH only treatment is effective in removing hemicellulose. The predominance of the hemicellulose signals shows that this treatment is effective in removing both hemicellulose and lignin.

#### X-Ray diffraction

The samples were submitted to X-ray diffraction and the diffraction data analysis was performed in order to calculate the crystallinity index of eucalyptus bark before and after pretreatments [51]. This analysis considers the relative intensities of the 002 peak for cellulose I and the minimum dip between the 002 and the 101 peaks, which are assigned to the amorphous region.

**Figure 7:**
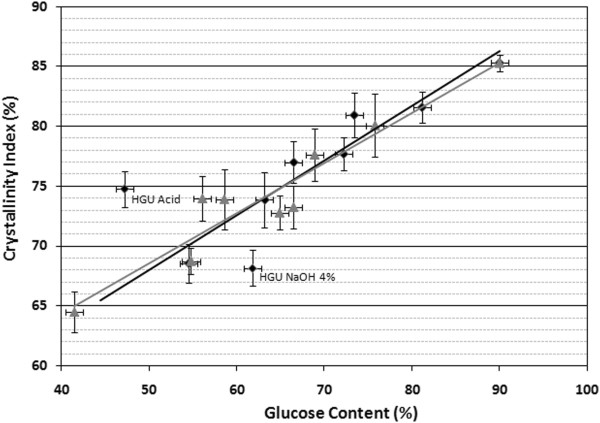
Determination of crystallinity index of HGU and EG eucalyptus clones bark, before and after different pretreatment condition.

Figure 7 shows the relative crystallinity index (CI) calculated for both eucalyptus barks as a function of their glucose percentages after different pretreatment conditions. The crystallinity index obtained from commercial sample that consists of approximately 90% crystalline cellulose (Avicel) is also shown for comparison.

The hot water extracted eucalyptus barks HGU and EG presented a crystallinity index of 68.6 ± 1.6% and 64.5 ± 1.7%, which corresponds to a glucose amount of 54.5% and 41.5%, respectively. The crystallinity of the HGU samples increased linearly together with the glucose content as the samples were treated with increasing NaOH, reaching a maximum CI of around 81.6 ± 1.3%, which corresponds to a 81.1% glucose content. A linear relation was also found between the CI of these HGU samples and the crystallinity index of the sample containing 90% of glucose (Avicel, CI = 85.3 ± 0.7%) [52]. Significant deviations from this linear behavior were observed in samples treated with 1% HCl or 4% NaOH only. The observed behavior for the sample treated only with acid can be explained by the fact that high glucose losses that took place, although the higher CI compared to the hot water treated material (73.9 ± 2.3%). The CI determined for the sample treated only with 4% NaOH (68.2 ± 1.5%) was very close to that found for hot water extracted HGU bark, which shows little or no alteration of the cellulosic fraction.

The crystallinity index found for EG bark is also shown in Figure 7. A linear correlation between glucose content and CI was found for all EG samples, reaching a CI = 80.1 ± 2.6% after treatment with HCl plus 4% NaOH (75.8 ± 6.8% glucose amount, Table 2). The sample treated only with 4% NaOH showed a CI and glucose amount similar to those found in samples treated with acid followed by 0.25% NaOH.

#### Scanning electron microscopy

**Figure 8:**
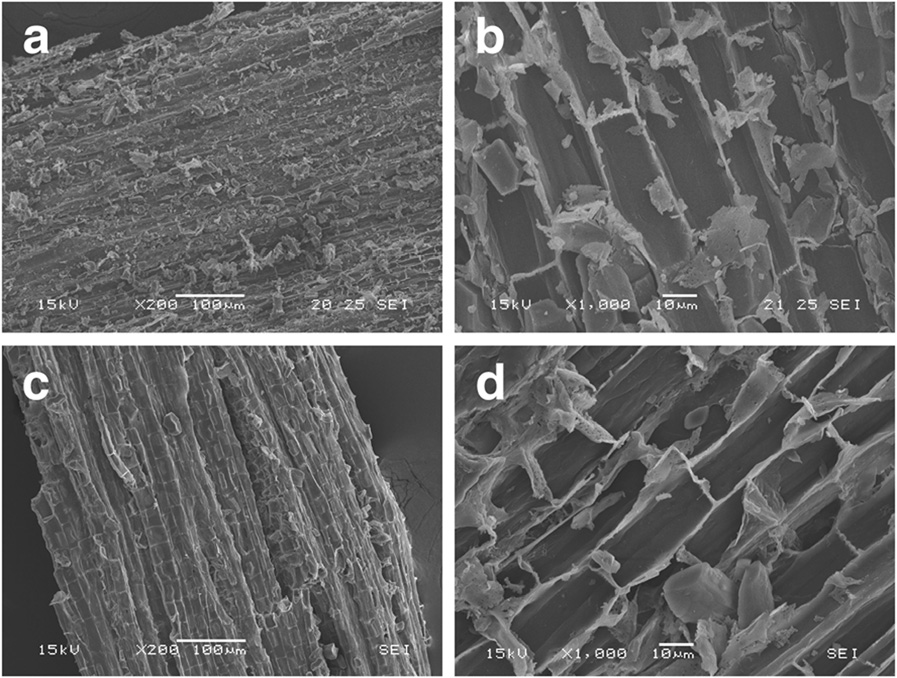
Surface images of eucalyptus bark obtained by SEM: (a) and (b) samples extracted with hot water only; (c) and (d) samples which underwent the acid treatment.

Modifications on the surface of EG and HGU eucalyptus bark samples caused by the pretreatments were analyzed using scanning electron microscopy. The observed effects on the fiber structure were very similar for both clones. Sample surfaces were imaged after soluble sugar extraction with hot water, and after acid and alkali pretreatments. Figure 8a-b shows the surface of a milled EG sample after hot water extraction, which reveals a tissue formed by ruptured cells covered with residues due to the milling process.

The total amount of residues on the sample surfaces is decreased after the acid treatment (Figure 8c-d). No other significant morphological changes could be observed. This is consistent with the notion that acid pretreatment mainly removes the hemicellulose fraction, which eucalyptus bark has a low content of. These images (especially Figure 8d) also show an important and distinctive characteristic of the EG bark radial section; namely the larger number of micropores at the boundary walls, which interconnect with neighboring cells, when compared to HGU bark.

**Figure 9:**
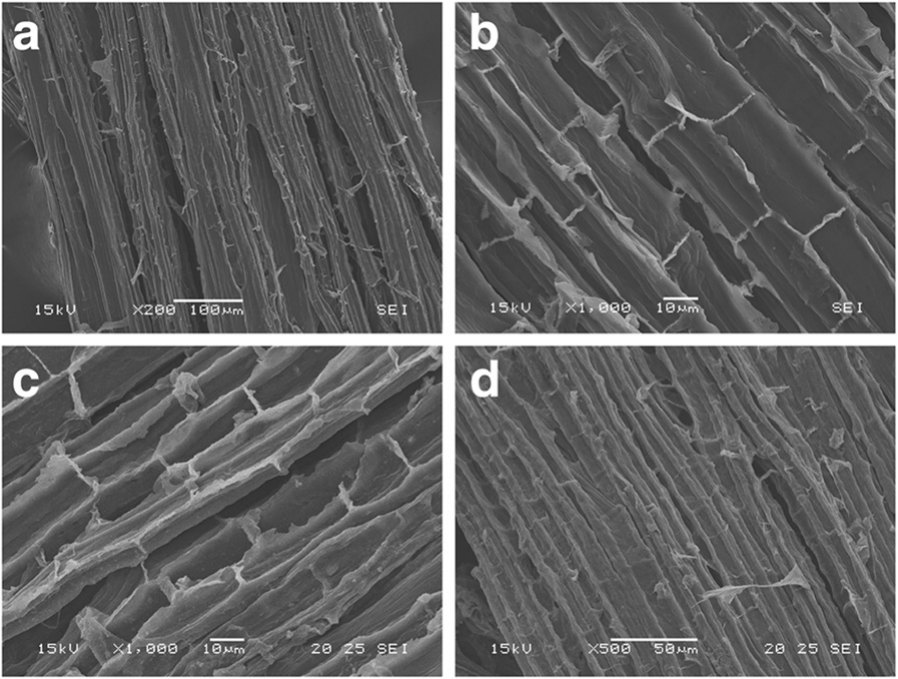
**Surface images obtained by SEM on eucalyptus bark samples treated different NaOH concentrations: (a) and (b) NaOH 0.5%; (c) and (d) NaOH 1.0%.** The amount of surface residues decrease with the alkaline treatment and neighboring cell bundles start to separate in the longitudinal direction.

**Figure 10:**
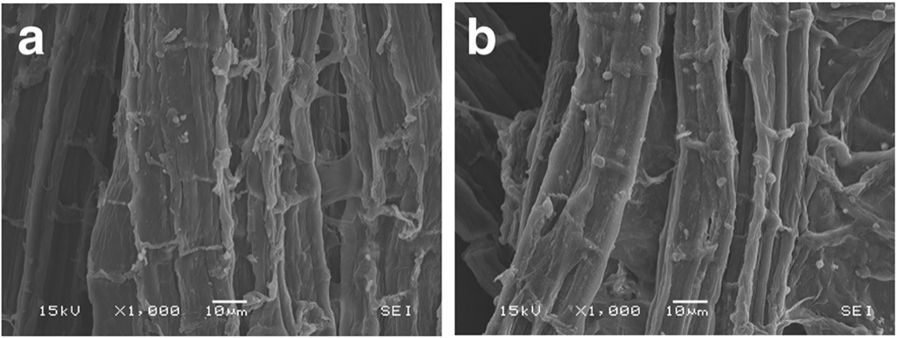
SEM images of eucalyptus bark samples treated with different NaOH concentrations: (a) NaOH 2.0%; (b)NaOH 4.0%.

Surface residues markedly decrease as samples undergo alkaline treatments (Figure 9a-d). In addition, the structures containing multiple pores at the boundaries of two cell walls are not visible after pretreatment. The most evident effect of alkaline pretreatment on the sample surface is the unidirectional separation of the cell wall bundles when NaOH concentrations above 0.5% are used (Figures 9 and 10). This structural change could be related to lignin removal as we observed that it is enhanced using NaOH concentrations higher than 0.5% (Table 2).

Increases in NaOH concentration, enhance the separation of the vascular bundles, which is more pronounced in samples treated with 2% and 4% NaOH (Figure 10 a-b). This is in agreement with the preferential localization of lignin in the middle lamellae, the membrane delimiting neighboring cells. Fromm and co-workers showed that more than 50% of the lignin is concentrated in the middle lamellae [53]. A correlation between the disaggregation of cell bundles and the lignin content of different biomasses has also been reported. The maximum separation of cell bundles produced on eucalyptus samples (using 4% NaOH) is less severe than the effect observed on sugarcane bagasse submitted to the same pretreatment conditions [29]. The higher cohesion between neighboring cell wall bundles after the alkaline pretreatment in the eucalyptus samples is probably related to the different lignin composition as well as the higher lignin content.

**Figure 11:**
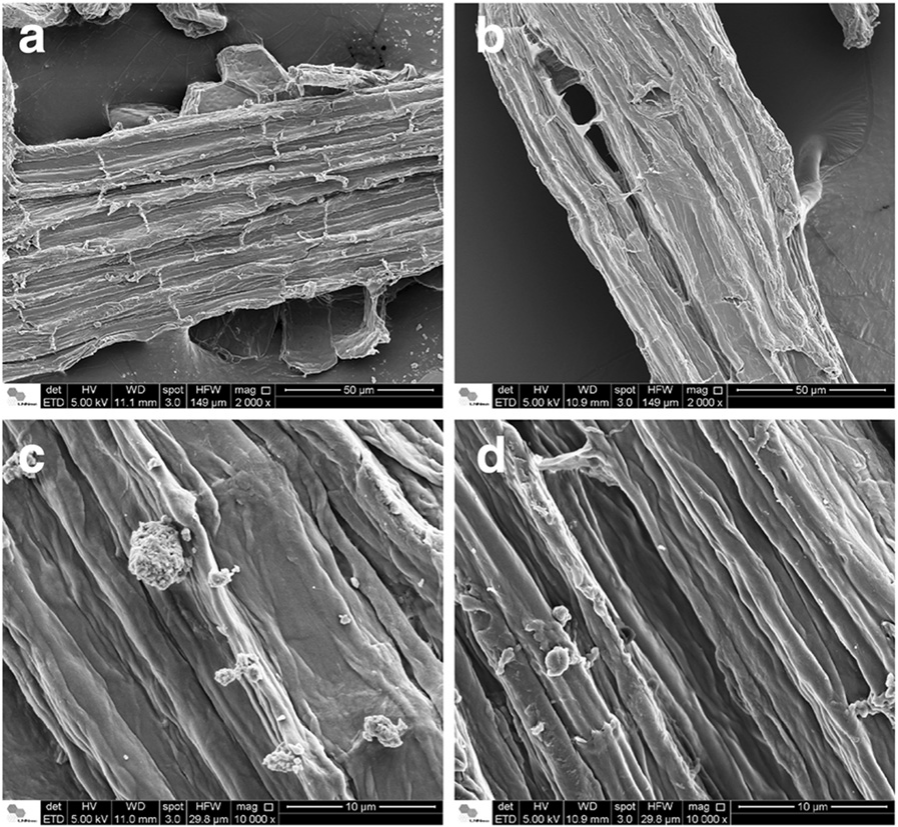
**SEM images of eucalyptus bark samples treated with 2.0% of NaOH.** (**a**) & (**c**): EG clone; (**b**) & (**d**): HGU clone. Globular structures assigned to lignin agglomerates are more frequently observed on EG than on HGU samples.

Another remarkable feature of these samples is the presence of globular structures associated to lignin condensation that were observed on the surface of EG and HGU barks after pretreatments with relatively high NaOH concentrations (2% or higher; Figures 10 and 11). The formation of lignin agglomerates on the surface has also been described in other lignocellulosic biomasses exposed to steam-explosion, diluted acid or organosolv pretreatments [54-57]. Previous studies showed that this phenomenon is related to the severity of pretreatment conditions. The lignin molecules become fluid and then coalesce, forming droplets within the cell wall matrix once the lignin phase-transition is reached (approximately between 120°C and 200°C) [54,56,57]. A fraction of this lignin is forced to the outer surface due to the hydrostatic pressures within the cell wall layers. The small droplets then come into contact with the pretreatment bulk and can deposit again on the biomass surface when cooling occurs [56].

In this study, the most lignin droplets were observed for bark samples treated with 2% NaOH and only a few droplets were seen on the surface of EG after 4% NaOH treatment. This probably occurred because the higher NaOH concentration (4%) was sufficient to keep most of the coalesced lignin dispersed in the pretreatment liquor and lower concentrations used (less than 1%) were not adequate to peel off the cellulose microfibrils and to expose the crystalline surfaces, which can adhere to each other by hydrogen bonding, causing the localized cell-wall collapse phenomenon that is pointed as the main driving force for lignin migration and extrusion [57]. EG shows a higher concentration of droplets on its surface when compared with HGU that has undergone the same pretreatment conditions (Figure 11). More than 20 areas were imaged in different particles of each sample treated under different conditions, in which this systematic tendency was confirmed: the amount and the frequency of lignin droplets are higher in EG than in HGU.

These differences on lignin migration and coalescence could be associated to lignin composition on samples bark, for example syringyl/guaiacyl (S/G) ratio. According to Barbosa *et al.* (2008), HGU wood samples submitted to analytical pyrolysis combined with gas chromatography/mass spectrometry (Py-GC/MS) showed a higher syringyl/guaiacyl (S/G) ratio when compared to EG wood [58]. The S/G ratio can directly affect the levels of energy, chemical and bleaching requirements for kraft pulp production. Higher contents of reactive S lignin allow for a much lower temperature and alkali concentration, shorter pulping time as well as less bleaching steps for processing hardwoods [59].

Previous studies showed that the presence of lignin droplets on the cell wall surface can have diverse effects upon the biomass enzymatic digestibility. Selig *et al.* (2007) observed a 10-20% reduction of glucose released from filter paper that had been impregnated by lignin droplets, which were extracted from maize by diluted acid treatment. However, no effect was observed when the same paper was impregnated by lignin from a hot water treatment [56]. The authors believe that the lower glucose release can be associated to a cellulose surface blockage, as well as to an increase in the nonspecific binding of cellulases to the exposed lignin surfaces. In both cases, the processes are clearly dependent on the pretreatment conditions, feedstock and enzyme preparation chosen. An opposite behavior was described by Koo *et al.* (2012), who observed an improvement in enzymatic digestibility due lignin migration and droplet formation [54]. According to them, the lignin changes and migration during pretreatment can greatly increase the enzymatic conversion in spite of enzymatic inhibition that might be caused by nonspecific cellulase adsorption, because the lignin deposition on the surface enlarges the sample pore volume and surface area.

#### Enzymatic hydrolysis

**Figure 12:**
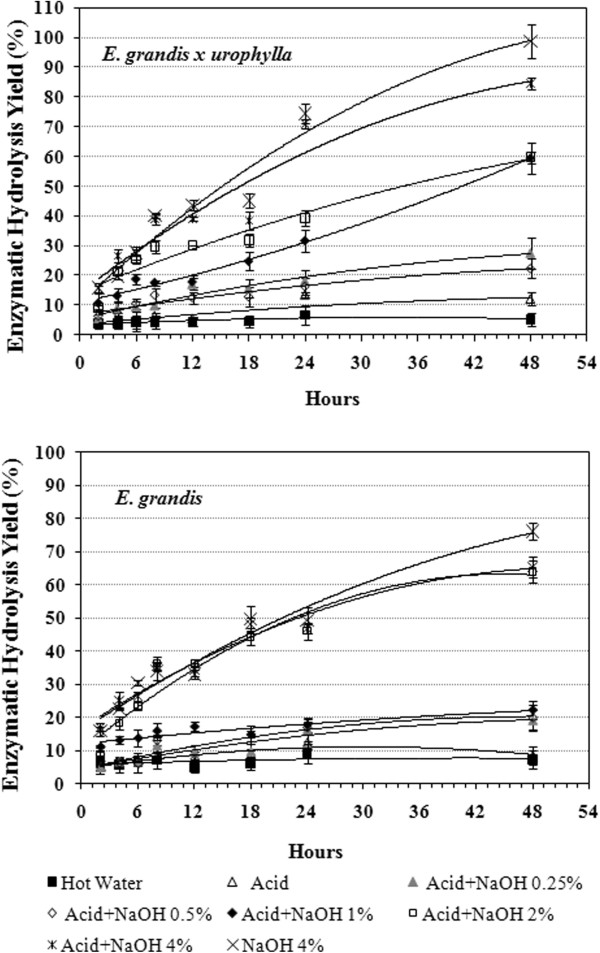
Enzymatic hydrolysis yield obtained for eucalyptus barks after acid and/or alkali treatments along 48 h.

The total amount of glucose released during enzymatic hydrolysis was measured to determine the potential of both eucalyptus barks before and after the different pretreatment conditions (Figure 12). The enzymatic digestibility of untreated bark and samples treated with diluted acid were quite similar for both barks, which is in agreement with our previous chemical and morphological analysis. Conversely, the barks digestibility was significantly improved by NaOH pretreatment. While only 5.2% and 7.4% of the available glucose was released from the hot water HGU and EG extracted samples, respectively, 84.5% and 65.4% of the glucose was released in samples treated with acid plus 4% NaOH after 48 hours of hydrolysis. In the HGU samples, the enzymatic digestibility was gradually increased with increasing NaOH concentrations, showing a significant difference between bark samples treated with acid plus2% or 4% NaOH, with approximately 60% and 85% of hydrolysis yield, respectively (Figure 12). EG samples showed a similar hydrolysis rate when treated with acid plus 2% or 4% NaOH, reaching around 65% hydrolysis efficiency. The high enzymatic digestibility of HGU bark can be associated to the lower concentration of lignin droplets observed on the cell wall surface, as revealed by SEM analysis. This difference can be explained by a higher solubilization of the coalesced lignin in the pretreatment bulk when 4% NaOH was applied, as no droplets were detected in HGU whereas EG contained some.

Surprisingly, the highest glucose release was found in both HGU and EG clones (98.6% and 78.5% respectively) when a single treatment step of 4% NaOH was analyzed. The single step alkaline treatment was effective in promoting enzyme accessibility to the cellulose chain through an increase in cell wall disorganization and separation. This was in spite of relatively higher residual lignin content and low hemicellulose removal when compared to samples treated with acid plus 4% NaOH. Also, the production of inhibitors, HMF and furfural (Table 2), from cellulose and hemicellulose degradation could be avoided if the acid step was not applied.

**Figure 13:**
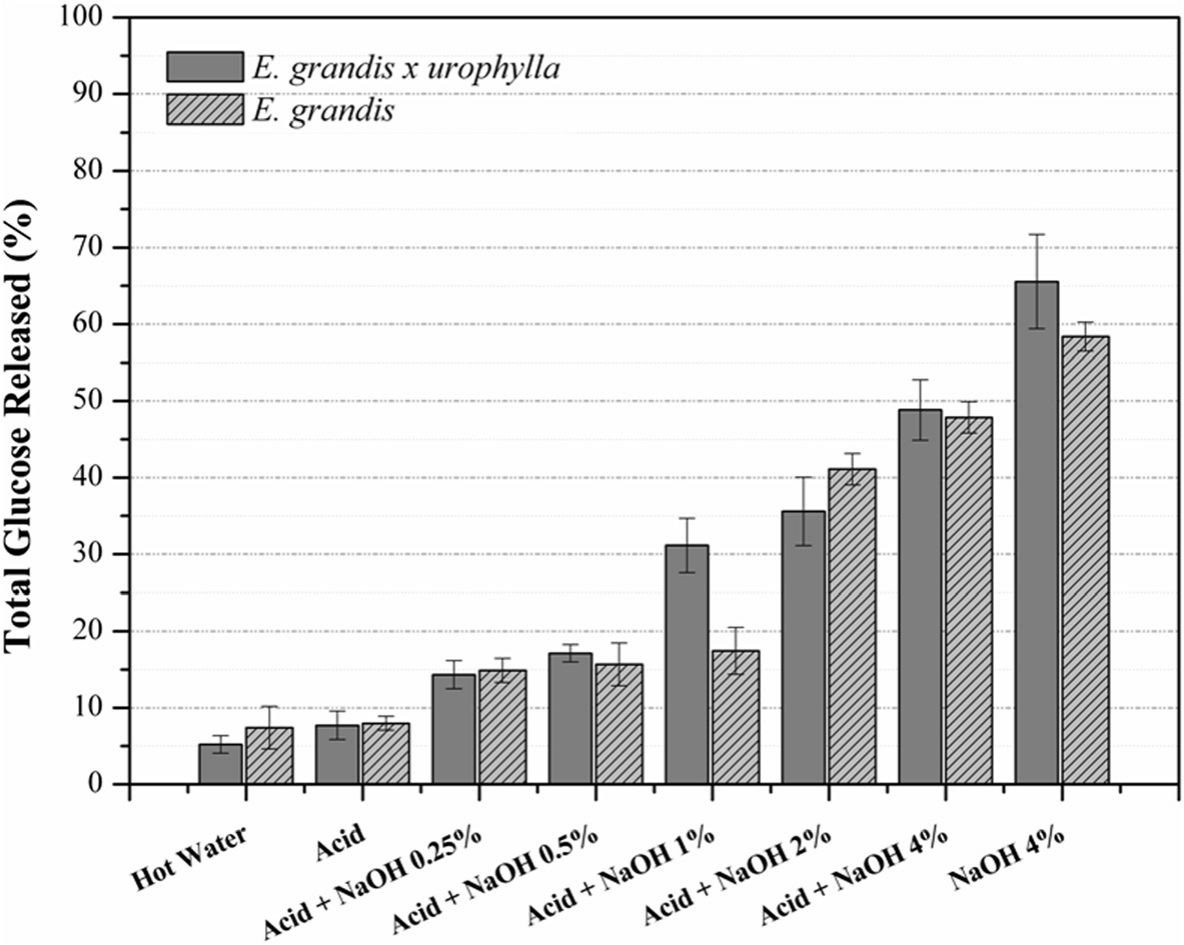
Total glucose released from both eucalyptus clones barks after 48 h hydolysis taking into account the losses during each pretreatment step.

The single step alkaline treatment with 4% NaOH also appears to be the best alternative when considering the glucose losses during the pretreatment steps (Figure 13). In spite of the high hydrolysis efficiency found for HGU (84.5%) and EG (65.4%), only around 50% of the initial glucose was released from the samples treated with acid plus 4% NaOH. This is a consequence of the high glucose losses, especially during the first acid pretreatment treatment step. Whereas, the single pretreatment with 4% NaOH releases about 65% of the initial glucose for HGU and 59% for EG due to lower losses during pretreatment.

## Conclusions

Our results underline the potential of eucalyptus barks, a poorly explored waste from paper and pulp, construction, and other industries, as a source of fermentable sugars for bioethanol production. The results in this paper indicate that eucalyptus bark is more susceptible to acid pretreatment when compared to sugarcane bagasse [29]. However, a single step alkaline pretreatment maximizes sugar yields. This represents an advantage in an industrial setting, because the enzymatic hydrolysis could still be efficiently performed, while the cellulose losses and the production of inhibitors of the enzymatic hydrolysis and fermentation steps during an additional acid pretreatment step were avoided. The higher amount and differences composition regarding of lignin composition in the bark, compared to grasses, demanded higher severity of in the alkaline treatment for lignin relocation than in grasses, for example sugarcane bagasse. Bark from the hybrid *Eucalyptus grandis x urophylla* (*urograndis*) showed a higher enzymatic digestibility when compared to *E. grandis*. This differential in saccharification can be associated to differences in lignin migration and coalescence on the cell wall surface, as well as to a higher S/G ratio. Barks from both eucalyptus clones showed potential to become a viable source of sugars for fermentation regardless of the differences in the hydrolysis yield. Indeed, the high C6/C5 ratio in eucalyptus bark makes this feedstock attractive for biochemical conversion into ethanol, as pentoses still remain a problem to the whole biorefinary process.

### Future research

Based on the present work, the total mass balance of an integrated process of ethanol production from eucalyptus barks will be developed, taking into account the fermentation yields from both total soluble sugars and the fermentable sugars obtained from the bark lignocellulose.

## Methods

### Plant materials

Barks from mechanized harvesting and stripping of wood, from the commercial clones *Eucaliptus grandis* (EG) and *Eucaliptus grandis* x *urophylla* (HGU) were kindly provided by Suzano Pulp and Paper Company (Itapetininga-SP/Brazil).

### Bark preparation

Eucalyptus bark was treated with hot water at 80°C for 1 h to remove the soluble sugars.

The soluble-free bark was dried in a convection oven at 60°C for 3 days, milled on a Wiley mill and passed through a 40–60 mesh sieve. The bark was then stored in plastic containers at room temperature and humidity until further use.

### Acid and alkali pretreatments

The eucalyptus barks were submitted to a two-step treatment process. The first step used diluted hydrochloric acid (HCl 1% v/v in water) followed by the second consisting of NaOH at a concentration between 0.25 and 4% w/v. The barks were pretreated with diluted HCl (1% v/v) for 1 hour at 120°C in an autoclave. The pressure was kept at 1.05 bar in a 1:10 solid to liquid ratio (gram of bark/ml of acid solution). After acid treatment the solid fraction was separated by filtration and thoroughly washed with water to remove any acid residues before oven drying at 60°C for 24 hours.

In the consecutive alkali pretreatment step the acid pre-treated barks were subjected to 0.25, 0.5, 1.0, 2.0, or 4.0% (w/v) NaOH at 120°C for 1 hour in an autoclave. The pressure was kept at 1.05 bar in a 1:10 solid to liquid ratio. Bark samples of each eucalyptus clone was submitted to an alkali treatment of 4.0% (w/v) without the previous acid pretreatment. Thus, a total of six different pretreated bark samples were obtained and were thoroughly washed to neutralize the pH. The solid fraction was then dried in an oven for 24 hours at 60°C.

All the samples were carefully weighed at the beginning and at the end of each treatment step to follow the mass balance. All the experiments were performed in duplicate.

### Chemical analysis

#### Determination of cell-wall components

Chemical composition of raw eucalyptus barks and pretreated samples were obtained by total acid hydrolysis with sulfuric acid (H_2_SO_4_), following previously described protocols [60]. The extractive fraction was removed by sequential extraction using toluol/ethanol (2:1 v/v); ethanol and water. After that, bark samples (100 mg) were treated with 1 mL 12 M H_2_SO_4_ solution, at 30°C for 1 hour, while vigorously stirring. Then, 28 mL of distilled water was added to the slurry, and the mixture was kept at 120°C and 1.05 bar for 1 hour to complete oligosaccharides hydrolysis. The hydrolysis reaction was stopped by fast cooling to 0°C. Samples were filtered and the liquid phases were analyzed by ion chromatography in order to quantify the sugar content. Monomer sugar (glucose, xylose, arabinose, galactose and ramnose) determination was performed in an ion chromatograph system (ICS 2500, Dionex, California), equipped with pulsed amperometric detection and a CarboPac™ PA1 anion exchange column, using a 5 mM NaOH solution as the mobile phase (flow rate 0.25 ml/min).

The soluble lignin was determined by absorbance at 280 nm using a UV–VIS Hitachi U-3300 spectrophotometer. The solid fraction was rinsed until neutral pH to remove acid residues and then oven dried at 105°C until constant weight (Klason lignin + Ashes) was achieved. Ash content was determined by burning in muffle at 800°C for 2 hours. Total lignin fraction was determined considering soluble and insoluble lignin fractions.

#### Liquor analysis: 2-furaldehyde and 5-hydroxymethylfurfural (HMF)

Liquor fractions obtained from each pretreatment condition were neutralized and chromatographed using a Luna® 5 μm C18(2) 100 Å LC Column 150 × 4.6 mm, together with C18 4 × 2.0 mm ID guard column (both from Phenomenex, Cheshire, UK) to verify furfural and HMF content. Analyses were carried out in a Surveyor HPLC (Thermo electron Corporation, Hemel Hempstead, UK), using an elution system of acetonitrile by reversed-phase in an isocratic gradient (5% acetonitrile and 95% deionized water) at 1 mL/min. The eluted furfuraldehydes were detected by UV absorbance at 284 nm using a Finnigan Surveyor PDA Plus detector. The 2-furaldehyde and HMF were quantified by interpolation of a calibration curve within the range of 0.005 ug/mL – 50 ug/mL of each standard in water.

#### Fourier transformed infrared spectroscopy (FTIR)

FTIR spectra of raw eucalyptus barks and samples collected after each pretreatment step were obtained at room temperature, using a Spectrum one FT-IR Spectrometer (PerkinElmer) equipped with an Attenuated Total Reflectance unit, at a wavelength setting ranging from 850 to 1850 cm^-1^. Measurements were done in triplicates and 256 scans were recorded for all samples at a 4 cm^−1^ resolution. Principal components analysis (PCA) was performed with Unscrambler X (CAMO ASA, Norway).

#### Solid state NMR

NMR experiments were performed on the eucalyptus barks of HGU and EG after hot water extraction and also on the solid and the liquor fractions resulting from pretreatments. The liquor fraction (hydrolysate) was prepared for analysis by neutralization followed by lyophilization.

Solid-state ^13^C nuclear magnetic resonance NMR experiments were performed using a Varian Inova spectrometer at ^13^C and ^1^H frequencies of 100.5 and 400.0 MHz, respectively. A Varian 5-mm magic-angle spinning MAS double-resonance probe head was used. Spinning frequencies of 5 kHz were controlled by a Varian pneumatic system that ensures a rotation stability of about 2 Hz. Ramped cross-polarization under magic angle spinning (CPMAS) combined with total suppression of spinning sidebands (TOSS) and heteronuclear ^1^H decoupling (CPMASTOSS) were used to acquire the ^13^C spectra. Typical π/2 pulse lengths of 3.5 μs (^13^C) and 4.5 μs (^1^H), cross-polarization time of 1 ms, acquisition time of 20 ms, and recycle delays of 2 s were used in all NMR experiments.

### Scanning electron microscopy (SEM)

Surface images from both eucalyptus barks after variable pretreatment conditions were analyzed by SEM and compared to the raw material. Milled samples were dried and coated with gold in a Balzers SCD 050 sputter coater. Sample imaging was carried out using a scanning electron microscope model JSM 5900LV (Jeol, Japan) and Quanta 650-FEG (FEI, USA) from the National Laboratory of Nanotecnology (LNNano-CNPEM) in Campinas-SP. A large number of images was obtained on different areas of the samples (at least 20 images per sample) to ensure the reproducibility of the results.

### X-Ray diffraction (XRD)

Raw eucalyptus barks and samples obtained from each pretreatment condition were analyzed by X-ray diffraction to evaluate their crystallinity index. XRD was performed in a Rigaku Rotaflex diffractometer model RU200B (Tokyo, Japan) using monochromatic CuKα radiation (1.54 Å) at 45 kV and 36 mA. Scans were obtained from 5 to 65 degrees 2θ (Bragg angle) at a 2°/minute scanning rate. Samples were milled prior to analysis and put through a 40–60 mesh sieve. The crystallinity index (CI) for all the samples were calculated according to the procedures previously described [46,51]. CI was obtained from the relationship between the height of the crystalline peak for cellulose (I_002_) and the height of the minimum (I_AM_) between the 002 and the 101 peaks, after background subtraction, according to Equation 1.

(1) $$\mathrm{CI}\left(\%\right)=\left[\left(I_{002}–I_{\mathrm{AM}}\right)/I_{002}\right]\times 100$$

The background signal was obtained measuring the empty sample holder under the same conditions used for the samples. A commercial sample containing 100% of microcrystalline cellulose (Avicel PH-101, Fluka) was also measured as a standard. Samples were measured in duplicates and the results presented are average values with their respective standard deviations.

### Enzymatic hydrolysis

Enzymatic hydrolysis assays were carried out at 50°C and pH 5.0, using 50 mM citric acid-sodium citrate buffer, in 250 mL Erlenmeyer flaks under a 200 rpm orbital agitation. Enzymatic hydrolysis experiments were performed at a solid to liquid ratio of 5% (gram of bark/ml of buffer solution), using an enzymatic cocktail that consisted of 25 FPU of Accellerase 1500 (Genencor, Denmark) supplemented with 12.5 BGU of beta-glucosidase from *Aspergillus nige*r (Novozyme 188; Novozymes, Denmark), per gram biomass.

The enzymatic hydrolysis yield (HY) was determined by considering the amount of released glucose (RG) in g/L and the glucose percentage (C) present within each of the bark samples, according to Equation 2 [61].

(2) $$\mathrm{HY}=\left[RG_{\left(g/L\right)}/\left(\mathrm{BC}\times C_{\left(\%\right)}\right)\right]\times 100\%$$

Where BC refers to the bark concentration (50 g/L). Assays were performed in duplicate and samples were collected at different hydrolysis time.

## Abbreviations

EG: *Eucalyptus grandis*; HGU: *Eucalyptus grandis* x *urophylla*; CP: Cross Polarization; CI: Crystallinity Index; DP: Dipolar dephasing; FTIR: Fourier transformed infrared spectroscopy; PCA: Principal component analysis; HY: Hydrolysis yield; MAS: Magic angle spinning; NMR: Nuclear magnetic resonance; SEM: Scanning electron microscopy; TOSS: Total suppression of spinning sidebands; XRD: X-Ray diffraction.

## Competing interests

The authors declare that they have no competing interests.

## Authors’ contribution

MAL planned conceptual process, performed the FTIR-PCA analysis, X-ray diffraction measurements, liquor analysis and was responsible for results analysis and manuscript draft. MAL, GBL and HKPS carried out the biomass pretreatment, enzymatic hydrolysis and chemical composition analysis. JB performed the initial characterization of raw barks. ERA and ODB performed the NMR experiments and analysis, and contributed to manuscript draft. CAR performed the SEM imaging and analysis and helped to draft the manuscript. LDG and SMM contributed to the FTIR-PCA and liquor analysis, as well as to the manuscript draft and language review. CAL and IP coordinated the overall study, and contributed to results analysis and writing up the paper. All the authors approved the final manuscript.
